# Mitochondrial Dynamics: Fission and Fusion in Fate Determination of Mesenchymal Stem Cells

**DOI:** 10.3389/fcell.2020.580070

**Published:** 2020-10-15

**Authors:** Lin Ren, Xiaodan Chen, Xiaobing Chen, Jiayan Li, Bin Cheng, Juan Xia

**Affiliations:** ^1^Hospital of Stomatology, Sun Yat-sen University, Guangzhou, China; ^2^Guangdong Provincial Key Laboratory of Stomatology, Guangzhou, China; ^3^Guanghua School of Stomatology, Sun Yat-sen University, Guangzhou, China

**Keywords:** mesenchymal stem cells, mitochondria, mitochondrial dynamics, mitochondrial fission, mitochondrial fusion, cell fate

## Abstract

Mesenchymal stem cells (MSCs) are pivotal to tissue homeostasis, repair, and regeneration due to their potential for self-renewal, multilineage differentiation, and immune modulation. Mitochondria are highly dynamic organelles that maintain their morphology via continuous fission and fusion, also known as mitochondrial dynamics. MSCs undergo specific mitochondrial dynamics during proliferation, migration, differentiation, apoptosis, or aging. Emerging evidence suggests that mitochondrial dynamics are key contributors to stem cell fate determination. The coordination of mitochondrial fission and fusion is crucial for cellular function and stress responses, while abnormal fission and/or fusion causes MSC dysfunction. This review focuses on the role of mitochondrial dynamics in MSC commitment under physiological and stress conditions. We highlight mechanistic insights into modulating mitochondrial dynamics and mitochondrial strategies for stem cell-based regenerative medicine. These findings shed light on the contribution of mitochondrial dynamics to MSC fate and MSC-based tissue repair.

## Introduction

Mesenchymal stem cells (MSCs) are multipotent stromal cells that originate from many connective tissues and can differentiate into a variety of cell types, such as osteoblasts, adipocytes, and myoblasts. MSCs are important for preserving tissue homeostasis and have regeneration potential (Bianco, 2014). As the progenitor cells of osteoblasts and osteocytes, MSCs can migrate to defective sites and initiate new bone formation during the early stage of bone healing (Tang et al., 2009). In addition to osteoblasts, MSCs are also able to differentiate into adipocytes within bone marrow microenvironment. Increasing evidence suggests that the differentiation of MSCs into adipocytes or osteoblasts is competitively balanced (Li et al., 2016), and this delicate balance is important for the maintenance of bone homeostasis (Hu et al., 2018b; Muruganandan et al., 2020). Dysregulation of osteo-adipogenic differentiation balance of MSCs contributes to development of bone diseases, such as osteoporosis, which manifests typically as a lineage shift toward adipocytes instead of osteoblasts in MSCs (Qi et al., 2017). Moreover, MSCs play a key role in the bone marrow microenvironment, which supports and regulates the stem cell niche and hematopoiesis (Wu et al., 2018). In recent years, MSCs have emerged as a promising tool for tissue repair and regeneration due to their multilineage differentiation potential, angiogenesis promotion, and immunomodulatory capacity (Chamberlain et al., 2007; Rajabzadeh et al., 2019). However, the effectiveness of MSCs is unstable since MSC fate is easily affected by the surrounding microenvironment, involving a complex regulation network (Sisakhtnezhad et al., 2017), and both endogenous and exogenous MSCs inevitably face harsh surrounding conditions and oxidative stress around the defected tissues (Chen et al., 2016; Sisakhtnezhad et al., 2017). Understanding the etiology of MSC dysfunction under stress and the underlying mechanisms would uncover unique avenues for novel and effective therapeutic strategies in MSC-based regenerative medicine.

Mitochondria are highly dynamic organelles that are key players in various biological processes in stem cells, including energy metabolism, oxidative stress reaction, calcium balance, and cell apoptosis. Dynamic changes in mitochondrial morphology are the basis for mitochondrial functionality (Chen and Chan, 2005; El-Hattab et al., 2018). Mitochondrial dynamics involves continuous fission and fusion, forming a dynamic network to maintain their abundance, morphology, and quality and cell function (Bereiter-Hahn and Vöth, 1994; Merz et al., 2007). This dynamic change in mitochondria can typically be simply characterized by morphological heterogeneity. Specifically, mitochondrial fission results in small and round mitochondria, while mitochondrial fusion leads to thin and elongated mitochondria with highly interconnected networks (Santel and Fuller, 2001; Stiles and Shirihai, 2012). Mitochondrial fission is essential for cell growth and division, providing sufficient numbers of mitochondria, sustaining cell polarity, and aiding in eliminating damaged mitochondria (Wai and Langer, 2016). In contrast, mitochondrial fusion allows for the exchange and connection of mitochondrial content, providing sufficient energy, alleviating oxidative damage, and maintaining membrane potential (Ikeda et al., 2015).

Although still in its infancy, emerging evidence indicates a pivotal role of mitochondrial dynamics in the self-renewal, differentiation, and death of MSCs. Mitochondrial dynamics are critical for MSCs to acquire the mitochondrial morphology required for specific behavioral needs, enabling cells to respond quickly and adaptively to environmental stresses. Intervention in mitochondrial dynamics can profoundly affect the MSC fate. In the current review, we illustrate the key mechanisms related to the posttranslational modification of mitochondrial dynamics proteins. Then, we reveal typical characteristics of mitochondria in MSCs and the contribution of mitochondrial dynamics to orchestrating MSC behavior under physiological and stressful microenvironments. Furthermore, we discuss the potential strategies for improving the therapeutic efficacy of MSCs by the modulation of mitochondrial dynamics.

## Regulatory Mechanisms of Mitochondrial Dynamics

Mitochondrial fission and fusion are orchestrated by a series of evolutionarily conserved proteins and dynamin-related GTPases. The large GTPase dynamin-related protein 1 (Drp1) and mitochondrial outer membrane receptors, including mitochondrial fission factor (Mff), mitochondrial fission protein 1 (Fis1) and mitochondrial dynamics proteins of 51 and 49 kD (MiD51 and MiD49), prominently control mitochondrial fission (Losón et al., 2013). Mitochondrial fusion is mainly mediated by optic atrophy 1 (Opa1) located on the mitochondrial outer membrane and mitofusin 1/2 (Mfn1/2) located on the inner membrane (Santel and Fuller, 2001; Chen and Chan, 2005). Here we list some protein kinase pathways that induce posttranslational modification of these proteins, especially phosphorylation, to further elucidate the regulatory mechanisms of mitochondrial dynamics (Table 1).

**TABLE 1:** Post-translational modification of some key factors involved in mitochondrial dynamics.

**Key factor**	**Post-translational modification**	**Effect on mitochondrial dynamics**	**References**
Drp1	Phosphorylation at Ser656 induced by active PKA	Reduced mitochondrial fission and swollen mitochondria	Cribbs and Strack, 2007
	Phosphorylation at Ser637 induced by active PKA	Reduced GTPase activity of Drp1 and impaired mitochondrial fission	Chang and Blackstone, 2007
	Phosphorylation at Ser600 induced by active PKA	Enhanced mitochondrial fission under norepinephrine treatment	Wikstrom et al., 2014
	Phosphorylate at Ser616 by induced by active ERK2	Enhanced mitochondrial fission	Kashatus et al., 2015
	Phosphorylation of at Ser579 induced by active ERK1/2	Enhanced mitochondrial fission in early stage of reprogramming	Prieto et al., 2016
	Phosphorylation at Ser616 induced by active p38 MAPK	Enhanced mitochondrial fission	Ko et al., 2017
	Phosphorylation at Ser616 induced by active AMPK	Enhanced mitochondrial fission	Li et al., 2019
	Phosphorylation at Ser616 induced by SIRT4 depletion	Increased Drp1 and Fis-1combination, enhanced mitochondrial fission	Fu et al., 2017
	Phosphorylation at Ser637 induced by active SIRT5	Reduced mitochondrial fission under starvation	Guedouari et al., 2017
Mff	Phosphorylation at Ser155 induced by active AMPK	Increased Drp1 recruitment, upregulated pSer616-Drp1, enhanced mitochondrial fission	Zheng et al., 2018
	Phosphorylation at Ser155, 172 induced by active AMPK	Increased Drp1 recruitment to mitochondria, enhanced mitochondrial fission	Toyama et al., 2016
Mfn1	Phosphorylation at Thr562 induced by active ERK2	Regulates Mfn1 oligomerization, increased Mfn1 combination with Bak, reduced mitochondrial fusion	Pyakurel et al., 2015
	Phosphorylation at Ser86 induced by beta II PKC	Partial inactivation of Mfn1 GTPase, increased mitochondria fragmentation	Ferreira et al., 2019
Mfn2	Phosphorylation at Ser442 induced by active PKA	Extensive perinuclear mitochondria	Zhou et al., 2010
	Phosphorylation at Ser27 induced by active JNK	Ubiquitin-proteasome degradation in Mfn2 and reduced mitochondrial fusion	Leboucher et al., 2012
	Ser-phosphorylation induced by active JNK	Mfn2 degradation and reduced mitochondrial fusion	Chakraborty et al., 2018
Opa1	Acetylation induced by cAMP/PKA mediated-degradation Sirt3	Proteolytic Opa1, inhibited mitochondrial fusion under tert-butyl hydroperoxide treatment	Signorile et al., 2017
	Deacetylation at Lys926 and 931 induced by active SIRT3	Enhanced mitochondrial fusion, sustain mitochondrial network	Samant et al., 2014
	Proteolytic induced by active SIRT4	Upregulation of L-Opa1, enhanced mitochondrial fusion	Lang et al., 2017
	Acetylation induced by SIRT3 deletion	Enhanced mitochondrial fission, dramatic mitochondrial fragmentation	Yi et al., 2019

### cAMP-Dependent Protein Kinase (PKA)

**FIGURE 1:**
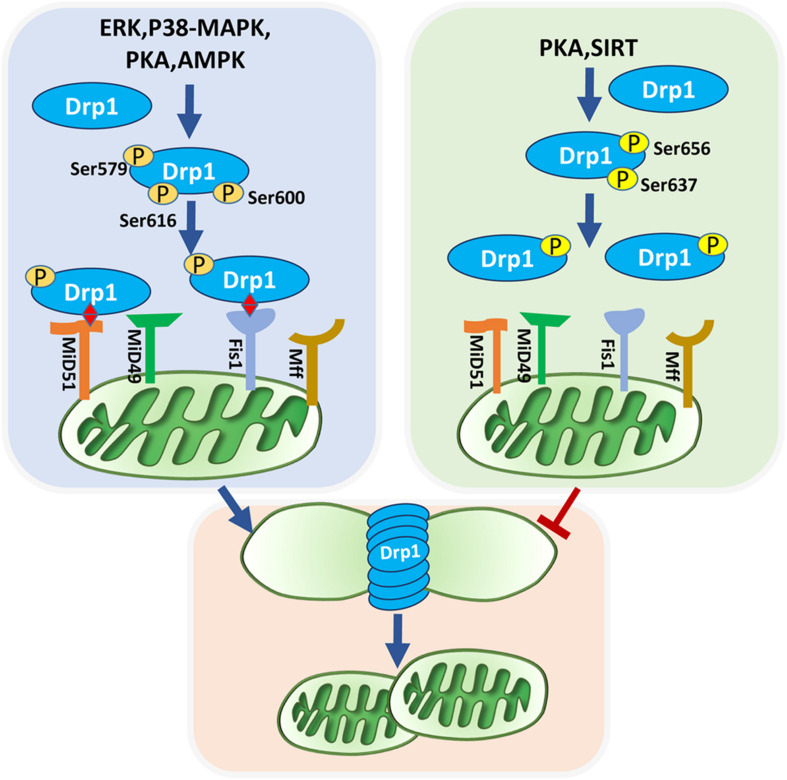
Phosphorylated Drp1-mediated mitochondrial fission. Mitochondrial fission is mediated by recruitment of Drp1 and their anchor on the outer mitochondrial membrane. Post-translational modifications of Drp1, especially phosphorylation, affect their localization in the cytoplasm or on the outer mitochondrial membrane. ERK, P38-MAPK, PKA, AMPK, and SIRT can phosphorylate Drp1. Phosphorylation of Drp1 at Ser637 and Ser656 inhibit mitochondrial fission, whereas phosphorylation of Drp1 at Ser616, Ser579, and Ser600 promote mitochondrial fission. Drp1 is anchored to the outer mitochondrial membrane via bounding with four receptors: Fis1, Mff, Mid49, and Mid51. Active Drp1 oligomers are assembled into ring-like structures that further constrict to sever the mother mitochondria into daughter mitochondria.

The ubiquitous second messenger PKA, one of the most well-investigated cytosolic kinases, is located on the mitochondrial surface and plays a regulatory role in maintaining mitochondrial activity, including mitochondrial dynamics (Feliciello et al., 2005; Cribbs and Strack, 2007). Drp1 relies on a GTP hydrolysis-dependent mechanism to form ring superstructures to contract and eventually incise mitochondria. Drp1 Serine 656 (Ser656) of Drp1 is a major PKA phosphorylation site in rat PC12 cells, this phosphorylation inhibits mitochondrial fission and reduces cellular sensitivity to apoptotic stimuli. Although Ser656 phosphorylation site is near the GTPase effector domain, there is no significant change in GTP hydrolysis among the Ser656 variants (Cribbs and Strack, 2007). However, in HeLa cells, active PKA suppresses Drp1 GTPase activity via Ser637 phosphorylation of Drp1, which may favor GTPase inactivation and Drp1 localization in the cytoplasm rather than recruitment to mitochondria, dampening mitochondrial fission (Chang and Blackstone, 2007; Yu et al., 2019). In contrast, phosphorylation of Drp1 at Ser600 is also dependent on adrenergic-stimulated PKA activation, inducing increased mitochondrial fragmentation and energy expenditure in brown adipocytes (Wikstrom et al., 2014). These findings suggest that cAMP/PKA controls mitochondrial fission principally via Drp1 modulation, and its effect on fission depends on phosphorylation sites and Drp1 transport. In fact, post-translational phosphorylation of Drp1 predominantly affects Drp1 activity. The different phosphorylated sites of Drp1 and they mediated-mitochondrial fission are demonstrated in Figure 1.

Some evidence has confirmed the role of the cAMP/PKA pathway in influencing mitochondrial fusion-related proteins. In myoblasts stimulated by reactive oxygen species (ROS), decreased mitochondrial cAMP/PKA signaling induces Sirt3 degradation/proteolysis, which in turn promotes the hyperacetylation of Opa1 and short Opa1 generation, consequently leading to hyperfragmentation of mitochondria and cell apoptosis (Signorile et al., 2017). Treatment with 8-Br-cAMP, an analog of cAMP, reverses the detrimental effect of Opa1 on mitochondrial dynamics, as well as cytochrome c release under oxidative stress in cardiac myoblast cells (Signorile et al., 2017). Although Zhou et al. (2010) verified that the specific PKA phosphorylation site Ser442 of Mfn2 effectively promotes Mfn2-mediated inhibition of vascular smooth muscle cell growth, these effects are thought to be independent of mitochondrial morphology and dynamics changes.

### Mitogen-Activated Protein Kinases (MAPKs)

Mitogen-activated protein kinases are serine-threonine kinases that transmit signals driven by cytokines, hormones, and other factors from the cell surface to the nucleus. At least three MAPK families have been characterized, c-Jun N-terminal kinase (JNK), extracellular regulated kinase (ERK1/2), and p38, which are involved in a wide variety of cellular functions, such as proliferation, apoptosis, and differentiation (Widmann et al., 1999). The most well-characterized MAPK with a confirmed regulatory effect on mitochondrial dynamics is ERK1/2 (Serasinghe et al., 2015; Cook et al., 2017). Oncogenic Ras treatment can activate ERK2 to phosphorylate Drp1 at Ser616, resulting in an increase in mitochondrial fragmentation. Both activation of MEK activity and elevated ERK phosphorylation lead to consistent trends in the expression of Drp1 (Kashatus et al., 2015). ERK suppresses mitochondrial fusion by phosphorylating Mfn1 at threonine 562 (Thr562), which favors Mfn1 binding to BAK and promotes their oligomerization and activation, subsequently facilitating cytochrome c release and apoptosis. This indicates the significant role of ERK-targeted Mfn1 in modulating mitochondrial morphology and apoptosis for mouse embryonic fibroblasts (Pyakurel et al., 2015). ERK1/2 activation participates in mitochondrial fission mediation during the early stage of induced pluripotent stem cell (iPSC) reprogramming by phosphorylating Drp1 at Ser579. Additionally, MEK inhibitor treatment inhibits mitochondrial fragmentation, while mutations in Ser579 of Drp1 successfully rescue the fission inhibition caused by MEK inhibitor application (Prieto et al., 2016). Similarly, treatment with the MEK inhibitor, PD325901, results in dramatic decline in both ERK and Drp1616 phosphorylation, effectively reversing mitochondrial fragmented phenotype in oncogenic Ras-induced embryonic kidney cells (Kashatus et al., 2015).

JNK activation is connected to Mfn2 but not Mfn1 turnover (Chakraborty et al., 2018). Leboucher et al. demonstrated that JNK mediated the phosphorylation of Mfn2 at Ser27 of sarcoma U2OS cells in response to cellular stress, which contributed to ubiquitin-proteasome degradation in Mfn2 and enhanced apoptosis (Leboucher et al., 2012). Ko et al. (2017) showed that p38 MAPK promoted Drp1-dependent fission in MSCs, and treatment with SB203580, a p38 inhibitor, reversed Drp1 phosphorylation at Ser616, leading to a decline in fragmented mitochondria.

### Adenosine Monophosphate-Activated Protein Kinase (AMPK)

AMPK is a conserved, redox-activated cellular energy sensor and regulator that is sensitive to AMP/adenosine-5′-triphosphate (ATP) stimulation. This kinase is activated during ATP consumption in response to stresses, such as low glucose, hypoxia, and ischemia (Carling, 2004). Reduced ATP levels induced by, for example, iron overload (IO), can activate AMPK in bone marrow mesenchymal stem cells (BMSCs), followed by Mff phosphorylation, further resulting in Drp1 translocation to mitochondria and fission enhancement, which partly contributes to BMSC dysfunction derived from IO patients with myelodysplastic syndrome (Zheng et al., 2018). The expression of p-Drp1 was downregulated while Mfn2 was upregulated in BMSCs after they were treated with compound C, an AMPK inhibitor, and cell senescence was therefore increased. These results indicated that mitochondrial fission activated by AMPK protected BMSCs from senescence (Li et al., 2019). At low ATP levels, AMPK-mediated fission is promoted due to Drp1 transport to the mitochondrial membrane stimulated by phosphorylation of Mff (Ducommun et al., 2015; Toyama et al., 2016). Consistently, stimulation of U2OS osteosarcoma cells with either electron transport chain inhibitor complexes or AMPK agonists resulted in significantly increased mitochondrial fragmentation (Toyama et al., 2016). AMPK-mediated mitochondrial fission is widely involved in cellular reaction to energy stress, and these mitochondrial fission events may serve as a trigger to initiating mitophagy to remove damaged mitochondria (Youle and van der Bliek, 2012; Toyama et al., 2016).

In contrast, activation of AMPK by a specific AMPK activator reversed the reduction in drug-induced mitochondrial fusion via upregulation of Mfn1, Mfn2, and Opa1, thereby sustaining hepatocyte viability (Kang et al., 2016). AMPK activation by AICAR, a known activator of AMPK, induced fused mitochondria and interconnected networks when adipose-derived mesenchymal stem cells (ASCs) from pericardial adipose tissue were differentiated into adipocytes (Abdul-Rahman et al., 2016). In addition, AMPK activated by metformin is able to suppress Drp1-dependent mitochondrial fission and alleviate oxidative stress, thus ameliorating atherosclerosis in diabetic mice (Wang et al., 2017b). However, mitochondrial fusion activated by AMPK is not always related to positive consequences. TGF-1 induces mitochondrial fusion through the AMPK signaling pathway, which induces aging of vascular progenitor cells isolated from patients with Marfan syndrome (He et al., 2019).

These findings suggest that AMPK activation can have opposing effect on mitochondrial dynamics, which possibly depend on cell types, bioenergetic status, and oxidative stress levels. The positive situation is that active AMPK sustains well-quality mitochondria at least partially via triggering mitochondrial fission and subsequent autophagy. When damage or stress is alleviated or removed, AMPK may support mitochondrial function by strengthening fusion rather than fission. Although AMPK-mediated mitochondrial dynamics have been found to be involved in some cellular damage processes, their relationship and detailed mechanisms have not been well studied.

### Sirtuin (Sirt) Family

The Sirt family is composed of highly conserved nicotinamide adenine dinucleotide (NAD^+^)-dependent deacetylases (HDACs). There are seven mammalian sirtuin subtypes (SIRT1 to SIRT7) that have different subcellular localizations and regulate a variety of cellular functions through posttranslational modifications of target proteins (Huang et al., 2010). Among them, SIRT3, SIRT4, and SIRT5 are located on mitochondria and can mediate the activity of some proteins related to mitochondrial dynamics (Huang et al., 2010; Samant et al., 2014; Lang et al., 2017).

SIRT3-mediated deacetylation of Opa1 at lysine 926 and 931 enhances Opa1 GTPase activity and sustains mitochondrial morphology, protecting cardiomyocytes from doxorubicin-induced cell death (Samant et al., 2014). Accurately, OPA1 appears to work in two different isoforms. Long membrane-bound form of OPA1 is responsible for mitochondrial fusion. But cleavage of long OPA1 inhibits mitochondrial fusion accompanied with generation of fission or mitophagy-associated short, soluble forms (MacVicar and Langer, 2016). SIRT4 expression facilitates mitochondrial fusion with an incremental increase in mitochondrial mass by inducing long Opa1 instead of short Opa1, further contributing to decreased mitophagy in HEK293 cell lines (Lang et al., 2017). In addition to promoting fusion, Fu et al. (2017) showed that SIRT4 may modulate mitochondrial fission in lung cancer cell lines by reducing Drp1 phosphorylation and diminishing Drp1 recruitment to the mitochondrial membrane by modulating MEK/ERK signal and interacting with Fis1. SIRT5-overexpressing C_2_C_12_ cells contain large and lengthened mitochondria that are uniformly arranged in the cytoplasm partly due to SIRT5-mediated upregulation of Mfn2 and Opa1, whereas SIRT5-silenced cells display small, round mitochondria that are distributed around the nucleus (Polletta et al., 2015). Consistent with these findings, SIRT5-mediated fusion promotion in mouse embryonic fibroblasts was also reported by Guedouari, and the results showed that elongated mitochondria and depressed autophagy depended on SIRT5 under starvation conditions (Guedouari et al., 2017). SIRT5 deletion increased the expression of mitochondrial dynamic protein of 51 kDa, Fis1, and pDRP1-S637, subsequently causing Drp1 activation and its translocation to mitochondria, thus promoting mitochondrial fission (Guedouari et al., 2017).

The available evidence suggests that Sirt family members located on mitochondria generally promote mitochondrial fusion and/or inhibit mitochondrial fission, and this effect is largely accompanied by a reduction in mitophagy or autophagy. It is hypothesized that fused mitochondria modulated by Sirt subtypes on mitochondria help alleviate stress injury and avoid damage caused by mitophagy.

## Morphological Characteristics of Mitochondria in MSCs

Mitochondria are the major source of energy in the form of ATP, which is produced through oxidative phosphorylation (OXPHOS). The process of OXPHOS and ATP generation occurs in the inner membrane of mitochondria (Gilkerson et al., 2003; Strauss et al., 2008). The shape of mitochondrial cristae and total mitochondria affect electron transport chains and protein complex production, which is significant for bioenergetic output (Beninca et al., 2014; Khacho et al., 2014). On the one hand, mitochondrial morphology is tightly linked to mitochondrial bioenergetics (Hoppins et al., 2007; Cogliati et al., 2013); on the other hand, it is also a reflection of ever-changing dynamics. Generally, well-developed, interlinked mitochondria with complex cristae structures tend to produce energy more efficiently than immature, spherical mitochondria because they have a larger surface area that can hold more intermembrane proteins (Zick et al., 2009). The fused or interconnected morphology of mitochondria is commonly found in metabolically active cells that depend on OXPHOS for energy production (Zhang et al., 2018; Fu et al., 2019). In contrast, cells that utilize glycolytic metabolism for energy production primarily have unfused spherical mitochondria (Seo et al., 2018; Zhang et al., 2018). Such immature mitochondria are metabolically less energetic and less polarized (Collins et al., 2002).

Mitochondrial morphology in embryonic stem cells (ESCs) and iPSCs is generally in an immature state featured by perinuclear-localization and fragmented, spherical, or punctate shapes (Folmes et al., 2011; Zhou et al., 2012). Similar mitochondrial characteristics have been observed in predominantly quiescent non-transplanted hematopoietic stem cells (HSCs) (Papa et al., 2018, 2019; Liang et al., 2020). These immature mitochondria are consistent with the energy state of stem cells, which usually depend on glycolysis as their primary energy source. Although MSCs have glycolysis-dependent energy metabolism and immature mitochondria with poorly developed cristae, the shape of their mitochondria is relatively more tubular than that in ESCs and iPSCs (Seo et al., 2018; Fu et al., 2019). ASCs have typical tubular mitochondria that form a robust mitochondrial network (Alicka et al., 2019). Additionally, more than two-thirds of ASCs display small globular and linear tubular structures (Li et al., 2015). Another morphological analysis of mitochondria showed general wiry or tubular mitochondria in BMSCs (Jin et al., 2018). Correspondingly, mitochondria-mediated fission contributes to immature mitochondrial morphology in BMSCs (Feng et al., 2019). These mitochondrial characteristics in MSCs are consistent with the metabolic level of MSCs in which mitochondria maintain low mitochondrial activity, low ROS lever and provide energy primarily through glycolysis (Fillmore et al., 2015; Hu et al., 2018a). However, embryonic mouse neural stem cells (NSCs) have increased mitochondrial lengths and relatively developed networks compared with ESCs or iPSCs, although these cells depend on aerobic glycolytic metabolism (Khacho et al., 2016).

It seems that most quiescent stem cells prefer glycolysis and have immature mitochondrial networks. However, this is a common situation, rather than a fixed and universally applicable criterion. The details referring to mitochondrial morphology and regulatory mechanisms vary among different types of stem cells. One should likely consider other factors beyond energy metabolism to impact mitochondrial morphology and dynamics. Additionally, based on the immature networks dominated by mitochondrial fission, it can be hypothesized that mitochondrial fusion-related factors remain in the low activation state. This does not mean, however, that mitochondrial fusion is not important. In contrast, male germline stem cells with simple and punctate mitochondria are sensitive to a block in fusion, and knockdown of mitofusin or Opa1 results in dysfunctional mitochondria and dyslipidemia (Senos Demarco et al., 2019). Additionally, deletion of Opa1 or Mfn1/2 destroys the structure of mitochondria and causes cell dysfunction in NSCs (Khacho et al., 2016). These findings suggest that both fission and fusion are indispensable for maintaining a normal mitochondrial morphology in stem cells.

Moreover, even the same types of stem cells undergo metabolic transitions and distinct mitochondrial dynamics due to different pluripotent states and differentiation fates. ESCs have been found to exhibit two stable but epigenetically distinct pluripotent states, named naïve and primed (Zhou et al., 2012; Sperber et al., 2015). Even though naïve ESCs contain under-developed mitochondria, they rely on bivalent metabolism and display a dynamic transition from glycolysis to OXPHOS according to demand (Zhou et al., 2012; Sperber et al., 2015). In contrast, highly glycolytic primed ESCs and epiblast stem cells manifest more mature mitochondria with well-developed cristae compared with naïve ESCs (Zhou et al., 2012; Sperber et al., 2015). With differentiation toward neural progenitor cells, iPSCs gradually form fused mitochondria with well-defined cristae, accompanied by a metabolic switch from glycolysis to OXPHOS (Lorenz et al., 2017). Lymphoid dominant HSCs have longer mitochondria compared with other hematopoietic populations, and Mfn2 is indispensable for HSCs to maintain extensive lymphoid potential (Luchsinger et al., 2016). Even many differentiated cells have more developed mitochondrial networks than stem cells (Cho et al., 2006; Lambertini et al., 2015), and this mitochondrial structure change may not be linear and may suffer complex dynamic changes. Mitochondria display considerable structural diversity in response to different physiological conditions.

Together, mitochondrial networks vary among different types of stem cells, distinct pluripotent states, and specific commitment fates (Table 2). Such variable mitochondrial morphology mediated by mitochondrial fission and fusion is rather sensitive to environment stimulation and is highly plastic.

**TABLE 2:** Mitochondrial morphology in stem cells and differentiated cells.

**Stem cell type**	**Mitochondria morphology**	**Morphological change**	**Differentiated fate**	**References**
Naïve embryonic stem cells	Rounded to oval mitochondria	Mitochondrial elongation	Cardiomyocytes	Zhou et al., 2012; Kasahara et al., 2013; Sperber et al., 2015; Wang et al., 2017a
Primed embryonic stem cells	Elongated mitochondria with well-defined cristae			
Induced pluripotent stem cells	Globular mitochondria	Mitochondrial elongation	Neural progenitor cells	Prieto et al., 2016 Fang et al., 2016; Lorenz et al., 2017
		Mitochondrial elongation	Neurons	
Neural stem cells	Elongated mitochondria	Mitochondrial fragmentation	Committed progenitors	Khacho et al., 2016; Ribeiro et al., 2019; Beckervordersandforth et al., 2017
		Mitochondrial elongation	Neurons	
Mesenchymal stem cells	Tubular mitochondria	Mitochondrial elongation	Adipocyte, osteoblasts	Alicka et al., 2019; Feng et al., 2019; Forni et al., 2016
		Mitochondrial fragmentation	Chondrogenic commitment	
Non-transplanted hematopoietic stem cells	Small and globular mitochondria	Mitochondrial elongation	Lymphoid commitment	Romero-Moya et al., 2013; Papa et al., 2018; Luchsinger et al., 2016; Liang et al., 2020
Transplanted hematopoietic stem cells	Elongated and swollen mitochondria			

## Mitochondrial Dynamics in MSC Function

**FIGURE 2:**
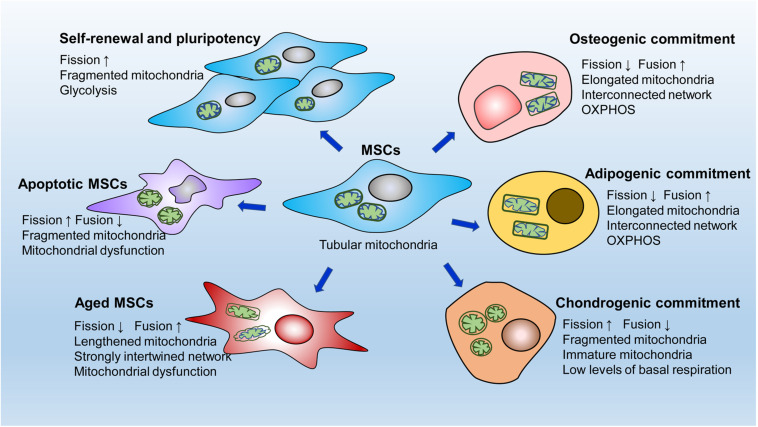
A simple diagram of mitochondrial dynamics in different MSCs behaviors. MSCs contain an immature mitochondrial network characterized by tubular mitochondria. Active mitochondrial fission, adapted to glycolytic dependence on energy production, is critical for the self-renewal and pluripotency of MSCs. After osteogenic or adipogenic induction, MSCs develop elongated mitochondria with interconnected networks. Correspondingly, MSCs undergo dramatic metabolic changes from glycolysis to oxidative phosphorylation for the energy supply. In contrast, in the early stage of chondrogenic commitment, fragmented mitochondria are clearly increased in MSCs accompanied by a low level of basal respiration. Mitochondrial fission is significantly enhanced in apoptotic MSCs, whereas mitochondrial fusion is markedly upregulated in aging MSCs.

Mitochondria undergo specific dynamic changes during stem cell proliferation, migration, differentiation, apoptosis, and aging (Figure 2). However, mitochondrial dynamics regulate cell fate by orchestrating the energy supply, intracellular ROS production and calcium balance.

### Mitochondrial Dynamics in MSC Pluripotency

As previously mentioned, fragmented mitochondria meet the energy needs of immature MSCs, exerting a fundamental role on intrinsic function (Seo et al., 2018; Zhang et al., 2018). Mitochondrial fission is essential for maintaining stemness in MSCs, and inhibiting fission leads to a reduction in the expression of stemness markers and multidirectional differentiation potential (Feng et al., 2019). During somatic cell reprogramming to iPSCs, cells undergo mitochondrial reconstruction, usually from a mature network toward immature mitochondria, and a metabolic shift, usually from OXPHOS toward glycolysis. Drp1-dependent mitochondrial fission is also necessary for the acquisition of cellular pluripotency during the early stage of embryonic fibroblast induction to form iPSCs (Prieto et al., 2016). Moreover, depletion of fusion-related Mfn1 and Mfn2 activates hypoxia-inducible factor 1α signaling, a necessary mediator of the metabolic switch to glycolysis, thus facilitating the transition in metabolic pattern from OXPHOS to glycolysis (Son et al., 2015). These processes promote the conversion of somatic cells to a pluripotent state (Son et al., 2015). However, over fission induced by Mff overexpression impairs the pluripotency of ESCs and iPSCs, suggesting that active fission should be within a certain threshold to better maintain stem cell function (Zhong et al., 2019). In summary, MSCs prefer mitochondrial fission to mitochondrial fusion to sustain the cell state and function, and active to-fission promotes the self-renewal and pluripotency of stem cells by stimulating glycolysis.

### Mitochondrial Dynamics in MSC Differentiation

In contrast to most stem cells, which have immature mitochondria, stem cells undergoing differentiation usually develop mature or specialized mitochondrial networks. MSCs undergoing differentiation display elongated and interconnected mitochondria accompanied by significantly decreased Drp1 and markedly increased OPA1 expression, indicating that mitochondrial fusion is conducive to MSC differentiation into adipocytes and osteocytes (Feng et al., 2019; Fujiwara et al., 2019). Similarly, Forni et al. induced osteogenesis and adipogenesis in MSCs and demonstrated enhanced mitochondrial biogenesis and network restructuring via mitochondrial fusion mediated by Mfn1 and Mfn2 during the early stages of induction (Forni et al., 2016). Furthermore, Mfn2 knockdown restrains respiratory activity, including basal ATP, maximal respiratory capacity, and H^+^-leak-related respiratory activity, leading to a loss of differentiation ability (Forni et al., 2016). The fused mitochondrial network facilitates mitochondrial energy production through OXPHOS, which corresponds to the energy required in these differentiated cells (Hsu et al., 2016; Li et al., 2017b). Consistently, during the differentiation of human iPSCs into cardiomyocytes, blocking Drp1 induces a metabolic transition from glycolysis toward OXPHOS, resulting in increased differentiation capacity in these cells (Hoque et al., 2018). Neuronal differentiation of iPSCs is closely related to upregulation of Mfn2 expression (Fang et al., 2016). Depletion of Mfn2 induces mitochondrial respiratory dysfunction by inhibiting complexes I and IV enzymatic activity and reducing ATP levels (Fang et al., 2016).

Interestingly, chondrogenesis in MSCs involves a fragmented mitochondrial phenotype at the beginning stage with increased expression of Drp1, Fis1, and Fis2 (Forni et al., 2016), which seems to contradict the finding that mitochondrial fusion is promoted during osteogenic and adipogenic differentiation of MSCs. Cells undergoing chondrogenesis do have low levels of basal respiration at the early commitment phase (Forni et al., 2016). Metabolomic analysis has revealed that catabolic processes occurring during the early stage of chondrogenesis are performed by both glycolysis and mitochondrial respiration (Kwon and Ohmiya, 2013). These metabolic changes differ from the enhanced mitochondrial OXPHOS that occurs during early adipogenic or osteogenic commitment (Chen et al., 2008; Li et al., 2017b), which may partly explain the increased fission to support glycolysis in MSCs during the early stage of chondrogenesis.

Mitochondrial dynamics clearly alter the differentiation fate of MSCs by regulating energy metabolism. However, mitochondrial dynamics-mediated regulation of MSC differentiation is not limited to energy metabolism regulation. Mitochondrial dynamics-dependent mitophagy may also be involved in modulating stem cell differentiation. Evidence has shown that mitochondria become fragmented before mitophagy enhancement (Forni et al., 2016; Marycz et al., 2016), demonstrating that mitochondrial fission is essential for mitophagy or autophagy (Gomes and Scorrano, 2008; Frank et al., 2012), which plays a critical role in MSC differentiation (Marycz et al., 2016; Vidoni et al., 2019). Additionally, although the relationship between mitochondrial dynamics and calcium homeostasis in MSCs has not been revealed, it has been suggested that mitochondrial dynamics can influence the differentiation of stem cells by regulating intracellular calcium balance. Zhong et al. (2019) reported that excess mitochondrial fission exacerbated cytosolic Ca^2+^ entry and CaMKII activity, resulting in the degradation of β-catenin and ultimately impairing the differentiation and embryonic development of iPSCs. Mfn2 negatively regulates calcineurin/NFAT activity by intracellular Ca^2+^ buffering, thereby maintaining HSCs with extensive lymphoid potential (Luchsinger et al., 2016). Kasahara et al. (2013) demonstrated that gene trapping of Mfn2 or OPA1 was sufficient to inhibit the differentiation of ESCs into cardiomyocytes. Mechanically, mitochondrial fusion orchestrates Ca^2+^ and calcineurin A to further affect Notch1-mediated suppression of the cardiomyocyte transition (Kasahara et al., 2013).

In short, although mitochondrial fission and mitochondrial fusion may exert pleiotropic effects during MSC differentiation depending on specific lineage commitment and different stages, we hypothesize that such mitochondrial dynamics undergo specific transformations to ensure the ever-changing energy demands and calcium balance, which is fundamental for the multidirectional differentiation of MSCs.

### Mitochondrial Dynamics in MSC Senescence

Long-term *in vitro* amplification is a common way that can induce senescence of MSCs (Kasper et al., 2009). After a certain number of cell divisions (7–12 passages), senescent cells increase, which is characterized by morphological abnormalities, enlargement, and increase of senescence-associated β-galactosidase positive cells. The long-term MSCs cultures (more than 100 passages) derived from rat have been found to exhibit increased susceptibility to senescence and have non-tumorigenic (Wagner et al., 2008; Geissler et al., 2012). Karyotype analysis in BMSCs reveals that aneuploidy chromosomal alterations may occurs during population doublings, but they became senescent without transformation features (Tarte et al., 2010).

Lengthened mitochondria often occur in various aging cells (Mai et al., 2010; Lin et al., 2015). Aged MSCs also exhibit a strong and complicated interconnected network that is distributed evenly in the cytoplasm, suggesting a potentiation of fusion processes (Geissler et al., 2012). p-Drp1 expression has been reported to be greatly downregulated, whereas Mfn2 expression is markedly upregulated in passage 12 (P12) BMSCs compared with those in P4 BMSCs, suggesting that these cells undergo aging accompanied by mitochondrial fusion (Li et al., 2019). Consistent with these observations, P7 ASCs have large tubular mitochondria forming an intertwined network that is regulated by Mfn1, Opa1, and Fis1 (Stab et al., 2016). In contrast, P2 ASCs show small tubular mitochondria forming a slightly interconnected network (Stab et al., 2016). Excessive mitochondrial fusion may adversely affect cells by altering ROS levels. Prolonged or giant mitochondria have been reported to augment ROS generation and weaken mitochondrial respiration activity in deferoxamine-induced senescent cells (Yoon et al., 2006). Furthermore, blocking mitochondrial fission, by overexpression of Drp1-K38A (active site is mutated in Drp1) and Fis1-ΔTM (transmembrane domain is deleted in Fis1), successfully leads to a senescent phenotype with ROS elevation in normal cells (Yoon et al., 2006). Additionally, the reduction in Drp1 levels during vascular aging exacerbates endothelial cell dysfunction by increasing mitochondrial ROS and suppressing autophagic flux, while the antioxidant *N*-acetyl-cysteine restores autophagosome clearance and improves angiogenesis in senescent endothelial cells (Lin et al., 2015).

Notably, increased mitochondrial fusion during aging is not always harmful (Stab et al., 2016). During fusion, depolarized mitochondria and normal mitochondria can join together to exchange their contents, further aiding in damaged mitochondrial repair and membrane potential maintenance (Twig et al., 2008b; Meyer et al., 2017). Of course, excessive fusion can be detrimental when depolarized and damaged mitochondria are overloaded, which is conducive to mitochondrial dysfunction during aging (Ikeda et al., 2015; Lang et al., 2017).

### Mitochondrial Dynamics in MSC Apoptosis

Conversely, cell apoptosis is usually accompanied by abnormal rupture of mitochondria in MSCs. Apoptotic ASCs isolated from equine metabolic syndrome (EMS) horses contain notably increased fragmented mitochondria (Kornicka et al., 2019). Analogously, prominently increased expression of Fis1 and decreased Mfn1 and Mfn2 expression have been reported in apoptotic human umbilical cord MSCs induced by monocrotophos exposure (Srivastava et al., 2018). Ma et al. (2019) demonstrated that dexamethasone increased mitochondrial fission and meanwhile diminished mitochondrial fusion. Mitochondrial fission was promoted by increased Fis1 and Mff expression, whereas the mitochondrial fusion was inhibited by decreased Mfn1 and Mfn2 expression. These mitochondrial dynamics alterations contributes to apoptosis enhancement and osteogenic suppression of BMSCs (Ma et al., 2019). Correspondingly, treatment with mdivi-1, an inhibitor of Drp1, dramatically ameliorated hydrogen peroxide (H_2_O_2_)-induced cellular apoptosis and death in human periodontal ligament stem cells (PDLSCs) and human W_8_B_2__+_ cardiac cells (Rosdah et al., 2017; He et al., 2018). Moreover, glucose/serum-deprived/hypoxia treatment-induced apoptosis in MSCs is reduced by increased glycolytic efficacy, which is modulated by the leptin/OPA1/SGLT1 signaling pathway (Yang et al., 2019).

The role of mitochondrial fission in modulating stem cell apoptosis is still unclear. Mitochondrial outer membrane permeabilization (MOMP) mediates the cascade conduction of many apoptotic signals. Arnoult et al. (2005) observed that Bax/Bak could facilitate the release of deafness dystonia protein 1 homolog, a member of mitochondrial intermembrane chaperone, into the cytoplasm, promoting its binding to the C-terminus of Drp1, this further results in Drp1 recruitment to mitochondria and Drp1-dependent mitochondrial fission. This Drp1-mediated mitochondrial fragmentation is vital for mitosis, which is involved in caspase-independent cell death (Arnoult et al., 2005). Earlier findings suggested that overexpression of Mfn1 reduces apoptotic HeLa cells induced by etoposide by impeding Bax transport to mitochondria and cytochrome c release (Sugioka et al., 2004). Intriguingly, other reports have shown that Drp1-mediated mitochondrial fission prevents cell apoptosis, especially Ca^2+^-related death (Szabadkai et al., 2004; Jahani-Asl and Slack, 2007). Specific upregulation of Drp1 leads to division of the mitochondrial network and damages the connectivity of the mitochondrial lumen, separating mitochondria from the endoplasmic reticulum, the source of calcium, which decreases Ca^2+^ absorption and ultimately abrogates Ca^2+^ overload-induced apoptosis (Szabadkai et al., 2004). However, these mitochondrial fission-dependent antiapoptotic events have not been demonstrated in stem cells.

Although current studies have shown that the apoptotic process of stem cells is dominated by mitochondrial fission, different apoptosis-inducing factors should be further studied to comprehensively understand the effect of mitochondrial dynamics on apoptosis. Overall, the interaction between mitochondrial dynamics and apoptotic signals is complicated. In response to different apoptotic or death pathway stimuli, mitochondrial dynamics may either support or restrain apoptosis. Increased apoptosis is usually attributed to enhanced mitochondrial fission since these processes act on Bcl-2 family dependent apoptotic pathways and related molecules. The effectiveness and mechanisms involved in mitochondrial dynamic-mediated alleviation of cell death caused by Ca^2+^ overload requires further confirmation.

## Mitochondrial Dynamics in MSCs Under Stress

Mitochondria can sense many stresses and influence cell survival and function by regulating a variety of signaling molecules. Mitochondrial dynamics play an important role in the mitochondrial stress response (Youle and van der Bliek, 2012; Meyer et al., 2017; Eisner et al., 2018). At present, the stress response of mitochondrial dynamics in MSCs primarily includes oxidative stress, metabolic stress, and some exogenous stimuli, such as physical stress and toxins.

### Mitochondrial Dynamics in MSCs Under Oxidative Stress

Oxidative stress occurs when the balance of the oxidative stress and antioxidant systems breaks down, which subsequently causes cellular damage. During physiological or repair processes, stem cells inevitably suffer attacks caused by oxidative stress. In fact, oxidative stress is a well-explored mechanism in regulating stem cell fate (Ko et al., 2012; Tan and Suda, 2018). Overgeneration of ROS indicates the occurrence of oxidative stress in tissues (Tan and Suda, 2018; Vina et al., 2020). Under pathological conditions, excessive ROS can be produced by mitochondria, which in turn leads to the inactivation of mitochondrial components (Zorov et al., 2014). The damaged members involved in mitochondrial dynamics may destroy the mitochondrial morphology and structure, resulting in a vicious cycle of continuous ROS release (Ježek et al., 2018). This reflects the complex interaction between mitochondrial dynamics and ROS generation.

Oxidative stress with ROS evaluation induced directly by H_2_O_2_ treatment leads to mitochondrial fragmentation in hMSCs; moreover, the combination of *N*-acetylcysteine, a biologic antioxidant, and ascorbic acid 2-phosphate, an oxidation-resistant derivative of ascorbic acid, successfully inhibits mitochondrial fission, decreases ROS production, and stabilizes mitochondrial membrane potential (Li et al., 2015). In another similar study of oxidative stress conditions stimulated by serum deprivation and hypoxia treatment, BMSCs had increased mitochondrial fragmentation along with upregulation of p-Drp1 Ser616 expression and downregulation of Mfn2 expression (Deng et al., 2020). In addition, an *in vitro* study underscored that CoCl_2_, a hypoxia mimetic, promoted mitochondrial fission in PDLSCs mediated by Drp1 elevation (He et al., 2018). Targeted inhibition of Drp1 markedly increased ATP levels, suppressed ROS generation, and eventually reduced cell apoptosis, indicating the important role of the ROS-Drp1-dependent mitochondrial pathway in CoCl_2_-induced apoptosis in PDLSCs (He et al., 2018). These findings suggest that high ROS levels and oxidative stress generally lead to abnormal mitochondrial dynamics, especially excessive mitochondrial fission. Reducing ROS levels helps to restore normal mitochondrial dynamics. Moreover, the regulation of mitochondrial dynamics can also be beneficial for reversing ROS overgeneration.

Unlike the high level of ROS, which is always associated with cell damage and disease, low or normal ROS level has been shown to have a positive effect on cell homeostasis and function via participating in signal transduction and promoting mitophagy (Shadel and Horvath, 2015; Palmeira et al., 2019). Early outbreaks of transient oxidative phosphorylation and elevated ROS in somatic cells promote NRF2 transcription factor activity, which further initiates the hypoxia inducible factor α-mediated glycolytic shift in early reprogramming (Hawkins et al., 2016). During reprogramming toward iPSCs, mitochondria undergo reconstruction dominated by enhanced mitochondrial fission, gradually forming an immature state instead of a mature mitochondrial network (Vazquez-Martin et al., 2012; Prieto et al., 2016; Lisowski et al., 2018). Compared with somatic cells, stem cells including MSCs have low ROS levels and immature mitochondrial networks (Hsu et al., 2016; Lisowski et al., 2018). Therefore, it is speculated that the changes in mitochondrial dynamics associated with low ROS levels are conducive to mitochondrial remodeling and adaptive changes.

### Mitochondrial Dynamics in MSCs Under Metabolic Stress

Studies on the effects of metabolic stress on mitochondrial dynamics mainly involve abnormalities in glucose and lipid metabolism. High levels of fatty acids alone or in combination with high glucose induce an increase in mitochondrial fragmentation (Molina et al., 2009). Dysfunctional ASCs isolated from patients with type 2 diabetes exhibit a similar trend in mitochondrial phenotype, in which overexpression of Fis1 causes fragmented, round mitochondria, and mitochondrial autophagy is also impaired, as indicated by reduced parkin RBR E3 ubiquitin protein ligase (PRKN, better known as Parkin) expression (Alicka et al., 2019). The Parkin is a crucial mitophagy regulator and its activation can build ubiquitin chains to ubiquitinating outer membrane protein on damaged mitochondria to label them for degradation in lysosomes (Bingol and Sheng, 2016). These findings are consistent with previous research by the same team, which showed that increased ASC apoptosis derived from EMS horses resulted in enhanced mitochondrial fission compared with the healthy control (Marycz et al., 2018). Similarly, metabolic syndrome impairs the swine ASC mitochondrial structure featured by an increase in mitochondrial fission and reduction of mitochondrial fusion (Farahani et al., 2020). Yu et al. (2006) have found that hyperglycemia-induced mitochondrial fission promotes ROS production and its periodic fluctuations, and either fission inhibition by Drp1 inhibition or fusion induction by Mfn2 overexpression prevents an increase in ROS induced by hyperglycemia.

### Mitochondrial Dynamics in MSCs Under Physical Stress and Toxins

Limited studies have indicated the potential impacts of physical stimuli or exogenous toxins on the mitochondrial dynamics of MSCs. Patten et al. (2019) observed that mitochondrial length in human BMSCs was slightly increased 4 h after exposure to 2 Gy radiation. They further demonstrated that Opa1 knockdown in mouse embryonic fibroblasts induced a decline in their adaptation to radiation, suggesting that mitochondrial networks may also be involved in the regulation of BMSC adaptation to radiation (Patten et al., 2019). Yin et al. (2017) revealed that low-level laser exposure facilitated mitochondrial biogenesis via upregulation of molecules associated with both mitochondrial fusion (Mfn1, Mfn2, and Opa-1) and mitochondrial fission (Fis1, Drp1, and MTP18), which contribute to elevated proliferation of BMSCs. In an *in vitro* study, methamphetamine exposure dampened the osteogenic differentiation of BMSCs due to abnormal OXPHOS and reduced ATP generation and mitochondrial membrane depolarization. These mitochondrial malfunctions were attributed to damaged mitochondrial biogenesis and mitochondrial fusion (Shen et al., 2018a). Similarly, treatment with carbon black Printex 90, a representative carbonaceous particle toxicant, induced mitochondrial dysfunction in BMSCs, which is closely related to suppressed mitochondrial biogenesis and mitochondrial dynamics, ultimately resulting in impaired osteogenic potential of BMSCs (Shen et al., 2018b).

### Multifaceted Effects of Mitochondrial Dynamics Under Stress

According to the literature, stress often results in fragmentation but not elongation events in mitochondria in MSCs, which is attributed to dramatic enhanced mitochondrial fission with/without inhibited mitochondrial fusion (He et al., 2018; Marycz et al., 2018; Shen et al., 2018a; Ma et al., 2019). In addition, the suppression effect on both mitochondrial fission and fusion was observed in BMSCs when they were treated with high levels of ferric ammonium citrate, a commonly used agent to induce iron overload (Yao et al., 2019). In contrast, Marycz et al. (2019) suggested that ASCs derived from metabolic syndrome horses displayed abnormal dysregulated and mixed mitochondria due to hyperactive mitochondrial fission and fusion. Moreover, stress-induced mitochondrial hyperfusion has been proposed by Tondera et al. (2009) as a particular prosurvival response to stress, and their research confirmed that exposure to a low dose of UV irradiation or actinomycin D efficiently induces hyperfusion of mitochondria in mouse embryonic fibroblasts within 9 h. Similarly, mouse embryonic fibroblasts show elongated mitochondria after starvation treatment, which protects them from autophagy-induced mitochondrial clearance (Gomes et al., 2011). Jendrach et al. (2008) observed that short-term and low doses of H_2_O_2_ result in a transitory increase in mitochondrial fusion in human umbilical vein endothelial cells. This enhanced mitochondrial fusion is beneficial to damaged mitochondria, in which mitochondrial DNA and membrane potential are sustained and the energy supply capacity is maximized to enhance adaptation under stress (Tondera et al., 2009; Gomes et al., 2011; Meyer et al., 2017).

**FIGURE 3:**
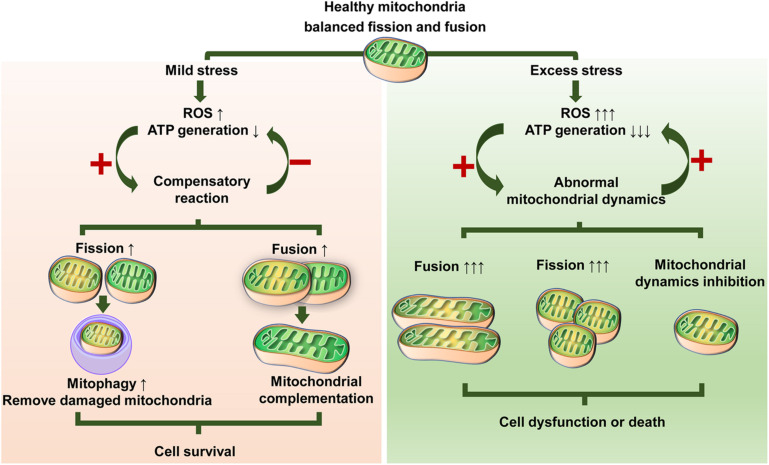
Illustration showing possible responses of mitochondrial dynamics to stress. Different stressors and stress levels lead to altered mitochondrial fission or/and fusion. Mild stress induces moderate ROS production and decreased ATP generation, which triggers adaptive changes in mitochondrial dynamics. Mitochondria can maintain their quality either by enhanced mitochondrial fission to remove damaged mitochondria or enhanced mitochondrial fusion to share components that, in turn, can relieve oxidative stress and fortify the energy supply, thus promoting survival. Excess stress causes a dramatic ROS increase and ATP exhaustion, which contributes to abnormalities in mitochondrial dynamics. In turn, abnormal mitochondrial dynamics exacerbate this situation via a vicious circle of continuous ROS elevation and/or ATP reduction, therefore inducing or worsening cell dysfunction or death.

Therefore, it is reasonable to conclude that different stressors and stress intensities can lead to complex changes in mitochondrial dynamics in MSCs, which are involved in fission and/or fusion activity. Mitochondrial dynamics seem to be central to bridging the gap between external stress and MSC function (Figure 3). Fusion events may act as a compensatory reaction in response to weak stress to better promote survival. In many cases, mitochondrial elongation is beneficial for resisting stress (Agarwal et al., 2016; Yang et al., 2019). However, upregulated fusion does not always mean elevated function. For example, senescent cells usually have a highly fused mitochondrial network, but their functions are relatively poor (Lin et al., 2015; D’Amico et al., 2019). Likewise, mitochondrial fragmentation is not always the result of maladaptation. Under stress states, impaired mitochondria may cause damage by ROS or other overgenerated harmful by-products and abnormal accumulation of Ca^2+^ (Shaughnessy et al., 2014; Park et al., 2017; Dilberger et al., 2019). Accordingly, timely and proper clearance of damaged mitochondria is imperative to cell survival and function. Mitophagy is the main way to selectively eliminate damaged or old mitochondria (Twig et al., 2008a; Ashrafi and Schwarz, 2013). The suppression of mitophagy impairs cell adaptability to stress and has been implicated in diabetes and neurodegenerative and cardiovascular aging diseases (Wallace, 2005; Egan et al., 2011; Marycz et al., 2016; Baechler et al., 2019). Mitochondria rely on mitochondrial fission to produce different daughter units. The daughter units with healthier membrane potentials will continue to participate in the dynamic cycle and facilitate recovery, whereas depolarizing mitochondria can be removed by mitophagy (Twig et al., 2008a). Inhibition of mitochondrial fission impairs mitophagy, while transitory enhanced mitochondrial fission acts as a protective strategy via cooperation with mitophagy (Twig et al., 2008a; Youle and van der Bliek, 2012). Stress most often induces hyperfission or abnormal mitochondrial dynamics, thus stimulating or worsening cell damage or death. Taken together, these findings suggest that the coordination of mitochondrial fission and mitochondrial fusion is a pivotal cytoprotective mechanism for cellular renovation and homeostasis under stress.

## MSC-Based Mitochondrial Therapeutics

The coordination of mitochondrial fission and mitochondrial fusion is essential for maintaining integrity and function of MSCs. Some crucial proteins that control mitochondrial dynamics play particularly important roles in regulating MSC fates. Based on these regulators, many strategies are being developed to guide mitochondrial dynamics to govern cell fate, thus improving the efficacy of MSC-based tissue repair.

Mdivi-1 is widely used as a selective Drp1 inhibitor that can inhibit the GTPase activity of Drp1. Some researchers have demonstrated the protective effect of mdivi-1 against apoptosis in stem cells under pathological or stress conditions. Targeted inhibition of Drp1 with mdivi-1 increases mitochondrial fission and protects human PDLSCs against H_2_O_2_-induced cell dysfunction (He et al., 2018). Mdivi-1 treatment rescues the EMS-induced abnormal mitochondrial network in ASCs, which subsequently reverses the senescence of ASCs, as indicated by suppression of p53, p21, and p62 expression (Kornicka et al., 2019). Moreover, short-term or intermittent administration of mdivi-1 is more beneficial for resisting tissue damage (Jheng et al., 2015). Single administration of mdivi-1 reverses the excessive fission of mitochondria and diminishes myocardial ischemia/reperfusion-induced damage in diabetic mice (Ding et al., 2017). Intermittent application of mdivi-1 1 and 16 h before insulin stimulation can improve insulin resistance in the skeletal muscles of obese mice (Jheng et al., 2012). Other mitochondrial fission inhibitors include P110, which blocks Drp1/Fis1 interactions, and dynasore, which non-selectively dampens GTPase activity. Several studies have reported that these inhibitors can prevent ischemia/reperfusion-induced damage in mouse hearts, suggesting their potential application for stem cells under oxidative stress conditions (Disatnik et al., 2013; Gao et al., 2013).

Gene modification and epigenetic regulation are also used to regulate mitochondrial dynamics and render MSCs more favorable for bone tissue repair. Silencing Mfn2 can diminish ROS levels and reverse the aging phenotype induced by deletion of the FGF21 in BMSCs (Li et al., 2019). Pretreatment of BMSCs with miR181-c activates the AMPK-Mfn1 signaling pathways, consequently reversing H_2_O_2_-induced negative effects on proliferation, migration, and paracrine activity in BMSCs (Fan et al., 2019). Mfn1 silencing abrogates the protective effect of miR-181c on BMSCs under oxidative stress conditions (Fan et al., 2019). Inhibiting miR-155-5p or Mfn2-siRNA reverses mitochondrial hyperfusion-mediated senescence in MSCs, and transplantation of aged MSCs pretreated with anti-miR-155-5p significantly ameliorates cardiac dysfunction in an infarction mouse model (Hong et al., 2020). miR-214 improves fibroblast differentiation of ASCs by directly binding to the Mfn2 3′-UTR, enhancing ASC-mediated repair in pelvic floor dysfunction in rats with birth trauma. Injection of a miR-214 inhibitor or overexpression of Mfn2 can counteract the therapeutic effect of ASCs in rat urinary tissues (Wu et al., 2017). Local application of lgr5-overexpression MSCs at the fracture site is reported as a superior method in augmenting bone healing in mice because of the positive regulation of mitochondrial dynamics and Wnt/ERK signaling pathways (Lin et al., 2019).

In addition to the targeted strategies described above, some compounds can also improve the biological activities of MSCs by modulating mitochondrial dynamics (Table 3). These studies suggest a complex regulatory network induced by mitochondrial dynamics that affects different MSC behaviors. Given the extensive and complicated role of mitochondrial dynamics, it is necessary to be more precise and prudent in exploring targeted regulatory strategies.

**TABLE 3:** A summary of compounds for mitochondrial dynamics modulation in MSCs.

**Compounds**	**MSC types**	**Mitochondrial dynamics effect and mechanism**	**Effect on MSCs**	**References**
Fibroblast growth factor 21	Human BMSCs	AMPK-Drp1↑ Mfn2↓ Mitochondrial fission↑	Senescence↓	Li et al., 2019
*N*-Acetylcysteine and Ascorbic Acid 2-Phosphate	Human ASCs	Drp1 S616 translocation↓ Mitochondrial fission↓	Mitochondrial function↑ mitoptosis↓	Li et al., 2015 Li et al., 2017a
Tyrphostin A9	Rat BMSCs	Mitochondrial fission↑	Stemness maintenance	Feng et al., 2019
Leptin	Human BMSCs	Opa1-mediated mitochondrial fusion↑	Survival in hypoxia↑ Glucose/serum-deprived/hypoxia-induced apoptosis↓	Yang et al., 2019 Yang et al., 2018
Haemin	Human BMSCs	p-Drp1 ser616↓ Mfn2↑ Mitochondrial fission↓	SD/H-induced apoptosis↓ Myocardial infarction-induced damage in mice↓	Deng et al., 2020
Succinate	Human MSCs	MAPK-P38p-Drp-1↑ mitochondrial fission↑	Migration↑ mice skin wound healing↑	Ko et al., 2017
PDGF-D	Human ASCs	p66shc-mediated mitochondrial fission↑	Migration↑ proliferation↑	Hye Kim et al., 2015
Icariin	Rat BMSCs	Fis-1↑ Mfn2↑ Drp1↑ mediate mitochondrial fusion and fission	Iron overload induced- osteogenesis inhibition↓	Yao et al., 2019
Pyruvate kinase muscle isoenzyme 2	Rat BMSCs	Drp1↑ Fis1↑ Mff↑ Opa1↓ Mfn2↓ mitochondrial fission↑	Osteogenic commitment↓ Adipogenic commitment↑	Guo et al., 2020
Melatonin	Mouse BMSCs	Opa1↓ Mfn1↓ mitochondrial fusion↓	Chronic kidney disease-related cellular senescence	Han et al., 2019

## Conclusion and Perspectives

Mitochondrial dynamics are closely related to the fate determination of MSCs. The coordinated collaboration of mitochondrial fission and mitochondrial fusion is of great significance in the self-renewal, multilineage differentiation, and stress response of MSCs. Proper regulation of mitochondrial dynamics benefits MSCs in sustaining viability, promoting differentiation, and resisting apoptosis, aging, and stress damage. Since mitochondrial dynamics vary with different MSC commitments and ways to participate in the response, and mediation of mitochondrial dynamics should be investigated in diverse MSC differentiation populations. In addition, the mechanism of mitochondrial dynamics in regulating the fate of stem cells includes energy metabolism alteration, oxidative stress modulation, and calcium homeostasis regulation. However, how these regulatory pathways mediate the interaction between mitochondrial dynamics and MSC behaviors requires further elucidation. Additionally, mitochondrial morphology and quality are integrally connected via mitochondrial biogenesis, fission, fusion, and mitophagy, and their crosstalk in MSC fates merits further exploration.

Stimuli such as oxidative stress often induce single changes in mitochondrial dynamics, especially fission activation, in which a strategy unilaterally targeting mitochondrial fission can achieve desirable results with reduced risk. However, the situation is sometimes complicated, and differentiation suppression in MSCs is accompanied by both abnormal fission and fusion (Shen et al., 2018b; Marycz et al., 2019; Yao et al., 2019). In such cases, changes in mitochondrial dynamics may involve a variety of dynamic-related proteins. Therefore, strategies targeting the mediation of mitochondrial dynamics will be complicated and risky and should therefore be carefully evaluated. It is also noteworthy that increased mitochondrial fragmentation may be due to enhanced fission and/or suppressed fusion, while elongated mitochondria are attributed to inhibited fission and/or intensified fusion. An accurate and comprehensive assessment is necessary to obtain the simplest but most effective strategy.

Although still at an early stage, modulating mitochondrial dynamics by various means to guide MSCs to better promote tissue repair is an emerging regulatory strategy that shows great potential in the future of regenerative medicine. More importantly, the definite crosstalk between mitochondrial dynamics and MSC fate should be clarified in detail before any conclusions can be drawn regarding how to direct mitochondrial fission or mitochondrial fusion toward controlling MSC behaviors.

## Author Contributions

LR was responsible for conceptualizing this review and writing the original draft. XDC was involved in the conceptualization, funding acquisition, and review and editing of the manuscript. XBC and JL participated in provision of resources and editing figures. BC contributed to funding acquisition, and participated in supervision, editing and revision of the manuscript. JX contributed to the editing, improving and finalization of the manuscript. All authors approved the final version of the manuscript and agreed to be accountable for this work.

## Conflict of Interest

The authors declare that the research was conducted in the absence of any commercial or financial relationships that could be construed as a potential conflict of interest.

## Abbreviations

MSCs: mesenchymal stem cells
Drp1: dynamin-related protein 1
Mff: mitochondrial fission factor
Fis1: mitochondrial fission protein 1
Opa1: optic atrophy 1
Mfn1/2: mitofusin ½
ROS: reactive oxygen species
MAPKs: mitogen-activated protein kinases
JNK: c-JunN-terminal kinase
ERK: extracellular regulated kinase
iPSCs: induced pluripotent stem cells
AMPK: adenosine monophosphate-activated protein kinase
IO: iron overload
BMSCs: bone marrow mesenchymal stem cells
ASCs: adipose-derived mesenchymal stem cells
OXPHOS: oxidative phosphorylation
ESCs: embryonic stem cells
HSCs: hematopoietic stem cells
NSCs: embryonic mouse neural stem cells
EMS: equine metabolic syndrome.
